# Supplementary material for: Formin 2 links neuropsychiatric phenotypes at young age to an increased risk for dementia
Source: EMBO J. 2017 Aug 2;36(19):2815–28. doi: 10.15252/embj.201796821 (PMC5623844; doi:10.15252/embj.201796821)
Supplement: Supplementary file 1 — Appendix [file EMBJ-36-2815-s001.pdf]

Appendix

Supplemental Methods and Materials

Behavior analysis

Mice were subjected to a series of behavioral tests as described before (*1*). Sample size for behavioral analysis was chosen on the basis of previous experiments and calculated to be have a power of 0,8. The experimenter was blinded. In brief, contextual fear conditioning was performed use the MedAssociates system using an automated tracking device. Fear conditioning consisted of a 3 min exposure to the conditioning context followed by one electric foot shock (2s, 0,7 mA, constant current). In the control experiment shown in supplemental figure 1D a lower foot shock intensity of 0.5 mA was used. For fear extinction learning mice were subjected to fear conditioning and extinction learning was analyzed by re-exposing mice (3 min) on consecutive days to the training context in a non-reinforced manner. Spatial memory was assessed using the water maze paradigm. The swimming path of the mice was recorded by a video camera and analyzed using Videomot 2 software (TSE). For reversal learning mice were first trained in the water maze paradigm as described above. Subsequently, the platform was moved to a new location before training was continued.

Explorative behavior and basal anxiety levels were assessed via the open field test. The explorative behavior of the animals was recorded by a video camera and analyzed using Videomot 2 software (TSE). The Rotarod test was performed in order to test motor function using the Rota Rod V4.02 System (TSE System). To test pattern completion in the water maze paradigm, animals were trained to find the hidden platform using all 4 visual cues. For the probe test 3 visual cues were removed and only one cue remained. For testing pattern completion in the fear conditioning paradigm mice were exposed mice on 3 consecutive days for 10 min to the conditioning context. On day 4 animals received an electric foot shock 10 s after being placed into the context. A memory test was performed 24h later and consisted of re-exposure to the conditioning context for 3 min.

Cannulation and siRNA injection.

Intra-hippocampal injections were performed as described previously (*2*) (*3*) (*4*) (*1*) In brief, mice were anesthetized and microcannulae were stereotactically implanted to the dentate gyrus (DG; 1.7 mm posterior to the Bregma; 1.0 mm lateral from midline; and 1.5 mm ventral). Mice were left to recover for at least one week prior to the start

of injections and behavioral experiments. After recovery from surgery, mice received bilateral injections Mice then received bilateral (1µl) injections of FMN2 siRNA (Santa Cruz, sc-45895; 250 µM) or scrambled siRNA (AllStars Negative Control, Qiagen SI03650318, dissolved in water at a concentration of 250µM) every 12h. For every injection, 10µl of mimic/mock were mixed with 1.35µl of HiPerfect reagent (Qiagen) as described in (*5*) and incubated at room temperature for 5min prior to injection For injections a 1.5mm-gauge needle that extended 0.5 mm beyond the tip of the guide cannula was used. The correct placement of the cannuale was verified at the end of the experiment via injection of methylene blue.

Immunoblot Analysis, co-immunoprecipitation, Immunohistochemistry & F/G actin assay

For immunoblot analysis crude lysates brain tissue was homogenized in TX buffer (50 mM Tris, 150 mM NaCl, 2m EDTA, 1 % Triton-X and protease inhibitors) incubated for 15 min at 4°C and centrifuged for 10 min (10000 rpm). The supernatant was used for immunoblotting. Immunoblots were performed using fluorescent secondary antibodies and data was quantified using an Odyssey Imager (Licor). Antibodies were diluted either in 0.5 % Milk PBT or 0.5% milk TBT. Subcellular fractionation was performed using the ProteoExtract Subcellular Proteome Extraction Kit (Calbiochem). Synaptosomes were isolated by homogenizing hippocampal tissue in 4 mL HEPES buffer (0.32 M sucrose, 4 mM HEPES; pH 7.6 adjusted with NaOH), 1 mM $MgCl_2$, 0.5 mM $CaCl_2$, 0.5 mM DTT, 1 mM EDTA.). The homogenate (H) was centrifuged at 800 × g for 10 min at 4 °C and the supernatant was again centrifuged at 9,200 × g for 15 min at 4 °C. The pellet was resuspended in 5 mL of HEPES buffer and used as crude snyaptosomes. Immunostaining was performed as described previously(*6*) and analyzed using a Leica SP2 confocal microscope. Alexa488 and Cy3 conjugated fluorescent secondary antibodies were purchased from Jackson Immunoresearch. The following antibodies were used in the mentioned concentrations: GAPDH, 1: 5000, Chemicon; Synaptophysin (Syp38) 1:1000, Sigma-Aldrich, Synaptoporin 1:200, Synaptic Systems, Formin 2 antibody was a gift from Philip Leder (*7*). ERK1/2, 1:1000. Santa Cruz, phosphor-ERK1/2, 1:1000, Sigma, phospho-SP1, 1:1000, Abcam, phosphor-ELK1, 1:1000, Santa Cruz For Fmn2 immunohistochemistry Fmn2-EGFP knock in mice were used, Cy3-labeled (goat anti-rabbit 1:500; Jackson ImmunoResearch, USA), Alexa488-labeled (donkey anti-mouse 1:500; Invitrogen, USA). For co-immunoprecipition target-specific antibodies

(see above) and non-specific control (IgG) were pre-coupled to surface-activated magnetic Dynabeads using an antibody coupling kit (life Technologies) at 20 µg of antibody per 1 mg of beads. After coupling beads were equilibrated with IP buffer (50mM HEPES, 100mM NaCl, 0.5% NP-40, pH 7.8, complete protease inhibitors (Roche). Protein lysate was incubated with the pre-coupled beads for 20 h. Elution was achieved by incubation of the antibody-antigen-bead complex with non-reducing SDS-PAGE loading buffer (1 M Tris-HCl, 50% Glycerol, 10%SDS, 5mM EDTA, 0,001% bromophenol blue for 10 min at 70). For analysis of the F-actin/G-actin In vivo Assay from Cytoskeleton, Inc. was used. The hippocampal dentate gyrus was isolated and processed accordingly in F-actin stabilization buffer. Analysis was performed according to the manual.

Electrophysiology

Mouse parasagittal brain slices of 14-21 days old FMN2 knockout mice and wildtype littermates were cut and stored up to 6 hours in a modified ACSF with partial substitution of sodium by sucrose (7). This solution was composed of (in mM) 87 NaCl, 25 NaHCO3, 25 glucose, 75 sucrose, 2.5 KCl, 1.25 NaH2PO4, 0.5 CaCl2 and 7 MgCl2 and was saturated with 95% O2/ 5% CO2. CA3 pyramidal cells were identified under infrared illumination with DIC optics and recorded in whole-cell voltage-clamp mode (3.5-4.5 MΩ electrodes, -70 mV holding potential) at room temperature. Slices were perfused with ACSF containing (in mM) 125 NaCl, 2.5 KCl, 1.25 NaH2PO4, 26 NaHCO3, 2.3 CaCl2, 1.3 MgCl2 and 25 glucose, saturated with 95% O2/5% CO2 and supplemented with bicuculline (10 µM) CGP55845 (1 µM). The intracellular solution contained (in mM) 140 Cs-methanesulfonate, 10 HEPES, 10 EGTA, 2 MgCl2, 2 NaCl and 4 Na2ATP, pH 7.3. Mf-EPSCs were elicited by randomly poking the dentate hilus with a patch pipette filled with extracellular solution until synaptic responses with the characteristic features of MFs (large paired pulse ratio and frequency facilitation) were found. The intensity of stimulation was then reduced to reach minimal stimulation conditions. Data was filtered at 2.9 KHz and digitized at 10 KHz. Series resistance was monitored throughout and recordings were discarded if it varied by >20%. Mossy fiber LTP was induced using a high-frequency stimulation protocol (HFS) consisting of 100 stimulations at a frequency of 100 Hz, repeated three times with a 10-sec interval between trains in the presence of bicuculline (10 mM) and the NMDA receptor antagonist D-AP5 (50 mM). HFS was performed at baseline stimulation intensity, which consisted of a single pulse delivered every 20 s (0.05 Hz). LTP was averaged between 20 and 30 min after the tetani. Mossy fiber LTD was induced by a long train of low frequency stimulation (900

pulses at 1Hz). The data were acquired using Pulse (Heka) and analyzed offline in Igor Pro 5 (Wavemetrics).

Gene expression

Library preparation and cluster generation for mRNA sequencing was performed according to Illumina standard protocols (TruSeq, Illumina). Libraries were quality-controlled and quantified using a Nanodrop 2000 (Thermo Scientific), Agilent 2100 Bioanalyzer (Agilent Technologies) and Qubit (Life Technologies). Data will be made available upon publication via GEO (accession number GSE100070). Base calling from raw images and file conversion to fastq-files was achieved by Illumina pipeline scripts. Subsequent steps included quality control (FastQC, www.bioinformatics.babraham.ac.uk/projects/fastqc/), mapping to reference genome (mm10, STAR aligner v2.3.0, (*8*) non-default parameters), read counting on genes or exons (HTSeq, http://www-huber.embl.de/users/anders/HTSeq, mode: intersection-non-empty) and differential gene (DESeq2_1.4.5 (*9*)) or exon (DEXSeq_1.10.8 (*10*)) usage biostatistical analysis. PCA and distance heatmaps were generated in R following instructions in the vignette for DESeq2. Genes were considered differentially expressed with an adjusted (Benjamini-Hochberg) as indicated in the figure legends. Gene set overlaps were calculated using Venny (http://bioinfogp.cnb.csic.es/tools/venny/). Gene Ontology (GO) functional analysis was carried out using Webgestalt unless otherwise stated (http://bioinfo.vanderbilt.edu/webgestalt/, (*11*)). For the analysis of FMN2 expression in hippocampal progenitor cells the multipotent, human hippocampal progenitor cell line HPC03A/07 (ReNeuron, Surrey, UK) was used to assess FMN2 expression change following dexamethasone (dex) treatment during neuronal proliferation and differentiation, similar to a previous study (*12*). Cells were either treated with 1uM of dex or vehicle during 3 days of proliferation only (3D, n=12) or for an additional 7 days of neuronal differentiation (10D, n=9). The FMN2 $\log_2$ expression levels were taken from an ongoing genome-wide analysis using HumanHT-12 v4 expression BeadChip arrays (Illumina) after extensive quality control as describe in (*13*). In addition, expression data was corrected for cell type heterogeneity computed using CETS package in R version 3.2.3 (http://www.r-project.org/) prior to differential expression analysis. Linear mixed models were used to assess the effect of dex on FMN2 expression array probe (ILMN_1764795) for each treatment with the biological replicates as random factor using the “lmer” function of the Lme4 package in R. P-values of these models were calculated using Wald test done by the “Anova” function of the package car in R.

ChIP analysis

ChIP was performed as described previously {Benito, 2015}. In brief, tissue was lysed in RIPA-SDS (140mM NaCl, 1mM EDTA, 1% Triton-X-100, 0.1% sodium deoxycholate, 10mM Tris-HCl, pH8, 1%SDS) buffer for 10min at 4C and then sonicated for 20min in a Bioruptor plus NGS (Diagenode). Chromatin was subsequently precleared by diluting it to 10x the initial volume in IP buffer (150mM NaCl, 1% NP40, 0.5% sodium deoxycholate, 50mM Tris-HCl pH8, 20mM EDTA, 0.1% SDS), adding 20µl of magnetic Dynabeads (Life Technologies) and incubating the mix at 4C for at least 4h in a rotating wheel. For H4K12ac ChIPs, 500µg of precleared chromatin and 0.5µg of anti-H4K12ac antibody (abcam Ref. ab61238) were allowed to interact overnight at 4C in a rotating wheel. IP'ed chromatin was then recovered by adding 15µl of magnetic Dynabeds and incubating further 2h at 4C in a rotating wheel. IP and input samples (input samples constituted 10% of the material used for ChIP) were collected and eluted in 20µl EB buffer (10mM Tris-HCl pH8) containing 0.1µg/µl RNaseA and incubated at 37C for 30min under gentle agitation in a Thermomix (Eppendorf). Samples were then further diluted ½ with WB buffer (100mM Tris-HCl pH8, 20mM EDTA, 2% SDS) and 1µl of PK (20mg/ml) was added. Samples were then incubated overnight at 65C under agitation in a Thermomix. DNA was recovered on the magnetic stand and re-eluted with EB buffer for 10min at 65C. Inputs were carried in parallel after the IP. DNA was precipitated with SureClean (Bioline) and linear polyacrylamide (LPA, Bioline), washed 2x with 70% EtOH and quantified using the Qubit (Life Technologies). Recovered DNA was used for qPCR.

SAHA administration.

SAHA was dissolved in drinking water following the recipe published in (*14*): 0.67g of SAHA (Cayman Chemical Company) was dissolved in 1L of drinking water containing 18g of b-cyclodextrin (Sigma, Ref 332607). The solution was heated to 95C and stirred until no SAHA particles were visible (approximately 20min) and then allowed to cool down to room temperature before making it available to animals. Animals drank on average 3 ml/day which equals 2 mg of SAHA intake per day. The maximum possible dose available to humans is 400mg/day. We used the following formular to covert the dose used in mice into human terms {Human Dose (mg/kg) = Rodent dose (mg(kg) x Rodent KM / Rodent KM / Human KM; KM Rodent = 3 and KM Human = 37} (*15*). Thus the dose applied to mice in this study approximates 5 mg/kg in humans. Taking into account an average weight of 75kg the dosage used

dose would equal 375 mg/day and is thus within the maximum tolerable dosage. For the experiments shown in Fig EV10 SAHA was injected directly into the hippocampus. Implantation of cannuale was performed as described for the RNAi experiments (see above). The total concentration of SAHA injected into the hippocampus was 50 µg.

Supplemental References

1. C. Kerimoglu *et al.*, Histone-methyltransferase mll2 (kmt2b) is required for memory formation in mice. *J Neurosci* **33**, 3452 (2013).
2. S. Peleg *et al.*, Altered histone acetylation is associated with age-dependent memory impairment in mice. *Science* **328**, 753 (2010).
3. A. Zovoilis *et al.*, microRNA-34c is a novel target to treat dementias. *EMBO J* **30**, 4299 (2011).
4. H. Y. Agbemenyah, R. C. Agis-Balboa, S. Burkhardt, I. Delalle, A. Fischer, Insulin growth factor binding protein 7 is a novel target to treat dementia. *Neurobiol Dis.* **25**, 135 (2013).
5. S. Bahari-Javan *et al.*, HDAC1 Regulates Fear Extinction in Mice. *J Neurosci* **32**, 5062 (2012).
6. R. C. Agis-Balboa *et al.*, A hippocampal insulin-growth factor 2 pathway regulates the extinction of fear memories. *EMBO J* **30**, 4071 (2011).
7. B. Leader *et al.*, Formin-2, polyploidy, hypofertility and positioning of the meiotic spindle in mouse oocytes. *Nat Cell Biol* **4**, 921 (2002).
8. S. Djebali *et al.*, Landscape of transcription in human cells. *Nature* **489**, 101 (2012).
9. W. H. M. I. Love, S. Anders, Moderated estimation of fold change and dispersion for RNA-Seq data with DESeq2. *bioRxiv*, (2014).
10. S. Anders, A. Reyes, W. Huber, Detecting differential usage of exons from RNA-seq data. *Genome Res* **22**, 2008 (Oct, 2012).
11. B. Zhang, S. Kirov, J. Snoddy, WebGestalt: an integrated system for exploring gene sets in various biological contexts. *Nucleic Acids Res* **33**, W741 (Jul 1, 2005).
12. C. Anacker *et al.*, Antidepressants increase human hippocampal neurogenesis by activating the glucocorticoid receptor. *Mol Psychiatry* **16**, 738 (2011).
13. A. Menke *et al.*, Dexamethasone stimulated gene expression in peripheral blood indicates glucocorticoid-receptor hypersensitivity in job-related exhaustion. *Psychoneuroendocrinology* **44**, 35 (2014).
14. M. Mielcarek *et al.*, SAHA decreases HDAC 2 and 4 levels in vivo and improves molecular phenotypes in the R6/2 mouse model of Huntington's disease. *PLos One* **6**, e27746 (2011).
15. S. Reagan-Shaw, M. Nihal, N. Ahmad, Dose translation from animal to human studies revisited. *FASEB J.* **22**, 659 (2007).

Supplemental figures

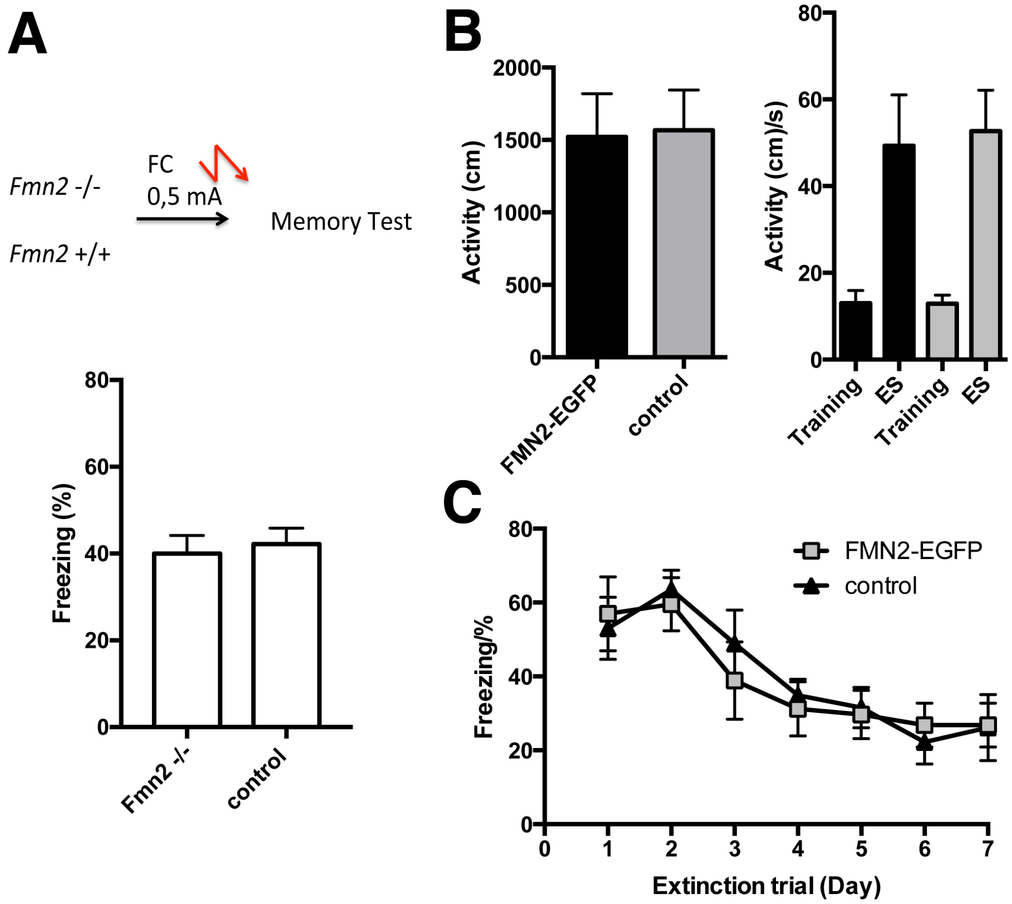

Appendix figure S1. FMN2 does not affect acquisition of fear memories. A. Upper panel: Experimental design. 3-month old *Fmn2 -/-* and control mice (n=10/group) were subjected to contextual fear conditioning using a mild electric foot shock (0,5mA, 2s). Lower panel: Freezing behavior was tested 24h later. No difference was observed amongst groups. **B.** To ensure that *Fmn2*-EGFP knock in mice (Fmn2-EGFP) could be used to study the localization of FMN2, we subjected *Fmn2*-EGFP mice to behavior testing relevant for our study. When subjected to fear conditioning training the activity during the training (left panel) and foot shock (right panel) was similar in *Fmn2*-EGFP and wild type control mice. These data indicate that explorative and behavior and pain sensation is normal in *Fmn2*-EGFP mice. **C.** Fear extinction learning was tested in *Fmn2*-EGFP and wild type control mice. Extinction learning was similar amongst groups. (n=8/group). Error bars indicate SEM.

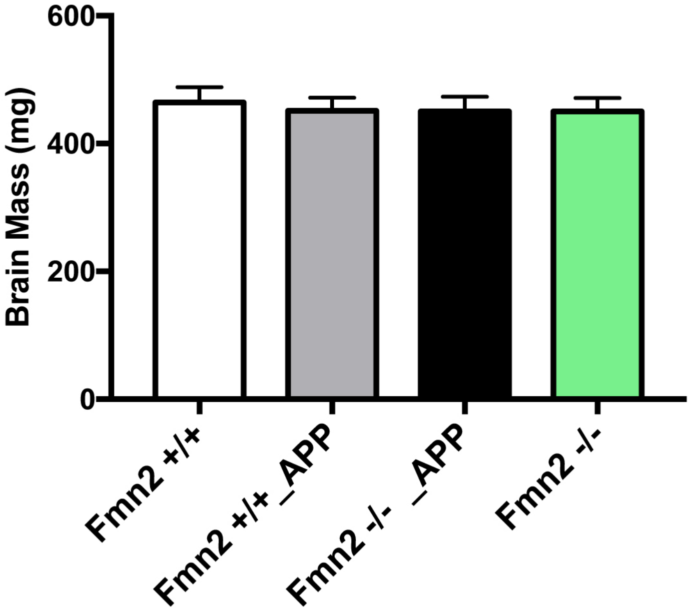

Appendix figure S2. Brain weight in *Fmn2*-/-_APPPS1-21 mice. Brain weight is similar amongst groups. (n=5/group). Error bars indicate SEM

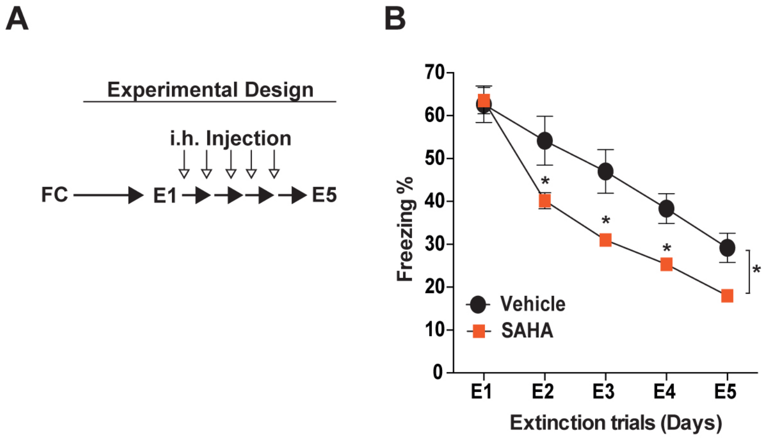

Appendix figure S3. The HDAC inhibitor Vorinostat (SAHA) facilitates fear extinction. A. Experimental design. Microcannuale were implanted into the dorsal dentate gyrus region of 3 month old male wild type mice (n=10/group). Mice were subjected to fear conditioning and showed similar freezing behavior when tested 24h later on E1. Immediately after each extinction trial mice were injected with either vehicle or SAHA (Vorinostat; 50µg). **B.** Intrahippocampal administration of SAHA after each extinction trial significantly facilitates fear extinction learning (*P<0,05 for within day comparisons; *P< 0,05 for repeated measurements).
