# Supplementary material for: Formin 2 links neuropsychiatric phenotypes at young age to an increased risk for dementia
Source: EMBO J. 2017 Aug 2;36(19):2815–28. doi: 10.15252/embj.201796821 (PMC5623844; doi:10.15252/embj.201796821)
Supplement: Supplementary file 2 — Expanded View Figures PDF [file EMBJ-36-2815-s002.pdf]

Expanded View Figures

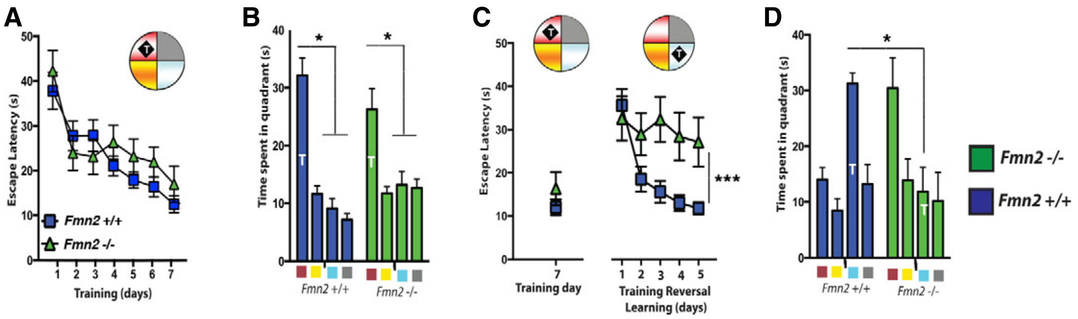

Figure EV1. Hippocampal FMN2 is required for reversal learning in the water maze paradigm.

- A Mice were trained in the Morris water maze test. Three-month-old *Fmn2*$^{-/-}$ and wild-type mice show similar escape latencies throughout training (n = 11/group).
- B A probe test performed after 7 days of water maze training revealed no significant difference amongst groups. Both groups showed a significant preference for the target quadrant (n = 11/group; *$P < 0.05$, t-test). The color key below the x-axis refers to the 4 quadrants used for the schematic water maze pool shown in panel (A).
- C Left panel: Three-month-old mice were subjected to water maze training until they could reliably find the hidden platform on day 7 of training. Right panel: Afterward, mice were subjected to reversal learning. Escape latencies during reversal learning were severely impaired in *Fmn2*$^{-/-}$ mice when compared to the control group (control, n = 15, *Fmn2*$^{-/-}$, n = 13; ***$P < 0.001$, F = 5.377, two-way RM ANOVA).
- D A probe test was performed after 5 days of reversal training. While wild-type mice showed a preference for the new target quadrant, target preference in *Fmn2*$^{-/-}$ mice was significantly impaired (control, n = 15, *Fmn2*$^{-/-}$, n = 13; *$P < 0.001$ versus *Fmn2*$^{+/+}$ control group, t-test).

Data information: T, target quadrant. Error bars indicate SEM.

© 2017 The Authors

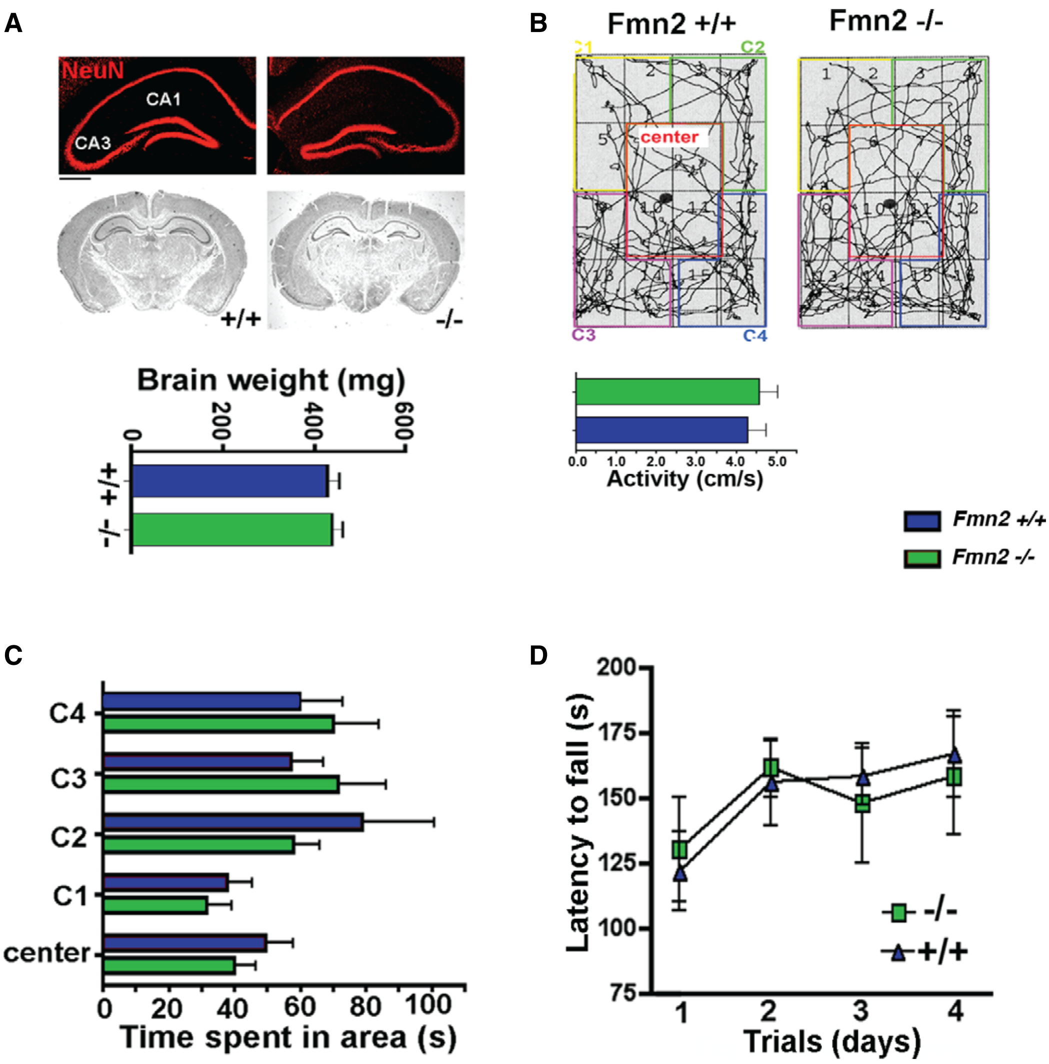

Figure EV2. FMN2 is expendable for locomotor function and basal anxiety.

A Upper panel: Representative images showing NeuN immunoreactivity in the hippocampus of 8-month-old *Fmn2*$^{-/-}$ and control mice. Scale bar: 200 μm. Middle panel: Silver staining showing that gross brain morphology is normal in 8-month-old *Fmn2*$^{-/-}$ mice. Lower panel: Brain weight is similar in 8-month-old *Fmn2*$^{-/-}$ and control mice (n = 5/group). CA1, hippocampal subregion CA1; CA3, hippocampal subregion CA3.

B *Fmn2*$^{-/-}$ mice show normal explorative behavior as assayed in the open-field test. Upper panel shows a representative image depicting the path in the open field. Lower panel shows the activity during the 5-min open-field exposure (n = 10/group).

C Basal anxiety as measured by the time spent in the four corners (C1–C4) versus the center of the open-field arena. No significant difference was found amongst groups (n = 10/group).

D *Fmn2*$^{-/-}$ mice show normal motor behavior in the rotarod test (n = 10/group).

Data information: Error bars indicate SEM.

 © 2017 The Authors

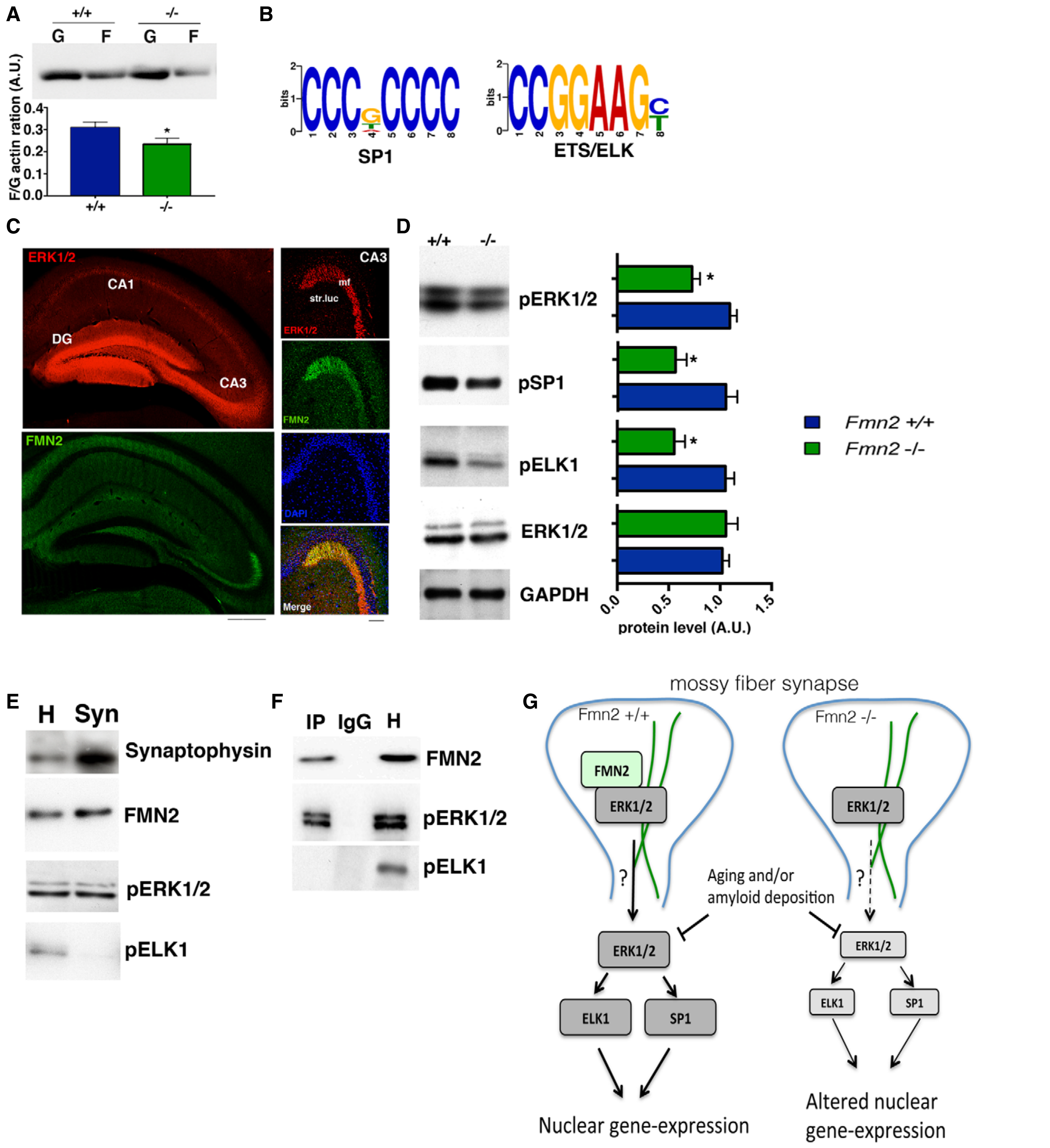
A
+/+
-/-
G F G F
F/G actin ration (A.U.)
+/+
-/-
B
SP1
ETS/ELK
C
ERK1/2
CA1
DG
CA3
FMN2
str.luc
mf
DAPI
Merge
D
pERK1/2
pSP1
pELK1
ERK1/2
GAPDH
protein level (A.U.)
Fmn2 +/+
Fmn2 -/-
E
H Syn
Synaptophysin
FMN2
pERK1/2
pELK1
F
IP IgG H
FMN2
pERK1/2
pELK1
G
mossy fiber synapse
Fmn2 +/+
Fmn2 -/-
FMN2
ERK1/2
Aging and/or
amyloid deposition
ELK1
SP1
Nuclear gene-expression
Altered nuclear
gene-expression

Figure EV3.

Figure EV3. FMN2 affects actin dynamics and ERK1/2-linked gene expression programs.

A FMN2 has been implicated with the regulation of actin dynamics and the actin cytoskeleton is essential for pre-synaptic plasticity and synaptic vesicle dynamics (Hatada *et al*, 2000; Owe *et al*, 2009). As such, we hypothesized that alterations to actin-related processes may contribute to the observed phenotypes. The levels of G and Factin were measured in the dentate gyrus isolated from $Fmn2^{+/+}$ and $Fmn2^{-/-}$ mice (n = 4) using the "F-Actin/G-actin In Vivo Assay" from Cytoskeleton, INC. The upper panel shows a representative image of the Western blot performed with the anti-actin antibody provided with the kit. The lower panel shows the quantification of the F/G ratio. There was a significant reduction in F/G-actin ratio in $Fmn2^{-/-}$ mice (*P = 0.0268, t-test).

B We analyzed the genes deregulated in 8-month-old $Fmn2^{-/-}$ mice for consensus motifs and target transcription factors at promoters of candidate genes using MEME-ChIP platform (Bailey *et al*, 2009; Machanick & Bailey, 2011) with default parameters. Targets with E-value above 0.05 were considered significant. Promoter regions were defined as ± 2 kb from TSS. We observed a significant enrichment of two motifs; "CCCGCCCC" which is a motif common for SP transcription factor binding sites and "CCGGAAGC" which is a target of ETS/ELK family of transcription factors.

C Representative images showing co-localization of ERK1/2 and FMN2 within the hippocampal mossy fiber pathway. Scale bars: 500 μm for low magnification images and 100 μm for high magnification images. mf, mossy fiber; str. luc, stratum lucidum.

D Immunoblot analysis showing decreased levels of active ERK1/2, SP1, and ELK1 that was measured using phospho-specific antibodies in the hippocampal dentate gyrus. Right panel shows quantification (n = 5/group; *P < 0.05; t-test).

E Immunoblot analysis of hippocampal synaptosomes (Syn) compared to cell homogenate (H), showing that FMN2 and pERK1/2 are present in the pre-synaptic compartment, while pELK1 is absent. Synaptophysin was used to confirm enrichment of synaptosomes (n = 3).

F Lysates from hippocampal synaptosomes were used for immunoprecipitation using an antibody detecting pERK1/2 (IP). IgG and homogenate (H) were used as negative and positive controls (n = 3). FMN2 and pERK1/2 but not pELK1 were detected in pERK1/2 immunoprecipitates (IP). These data indicate that FMN2 and pERK1/2 interact at synapses. As expected from our findings showing that pELK1 is not detected at synapses, we could not detect interaction of synaptic pERK1/2 and pELK1.

G A model suggesting how synaptic FMN2 may impact on gene expression. FMN2 and ERK1/2 interact at mossy fiber synapses (green lines indicate actin cytoskeleton). By mechanisms that remain to be determined FMN2 contributes to the activation of ERK1/2 that subsequently activates ELK and SP1 transcription factors which translocate to the nucleus and initiate gene expression. Of course we cannot exclude that FMN2 also affects gene expression by other mechanisms. In the absence of FMN2 (as in $Fmn2^{-/-}$ mice), the described pathway is less efficient which eventually contributes to altered gene expression. The fact that reduced ERK1/2 signaling is observed in the aging brain and in APP mice may explain why loss of FMN2 accelerates age-associated changes in gene expression.

Data information: Error bars indicate SEM.

 © 2017 The Authors

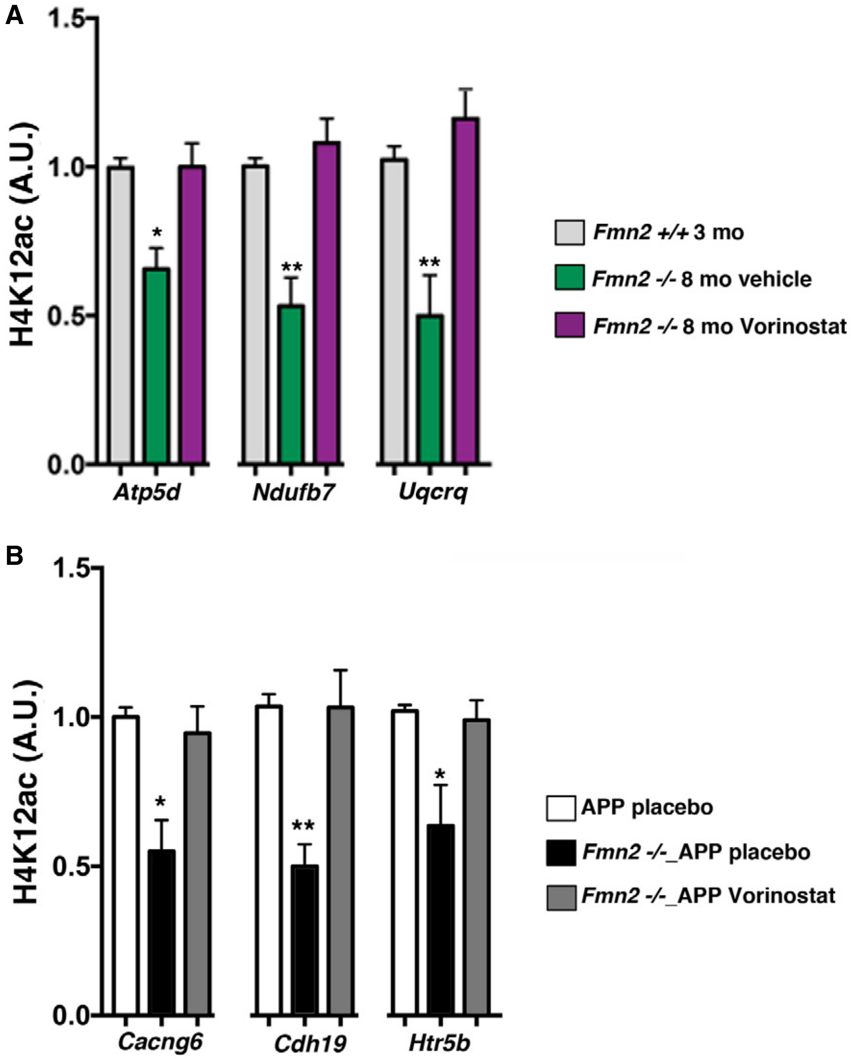

Figure EV4. Impaired H4K12ac in aged *Fmn2*$^{-/-}$ and *Fmn2*$^{-/-}$_APP mice is rescued by Vorinostat treatment.

A Quantification of ChIP-qPCR analysis showing H4K12ac at the promoter regions of three selected genes that showed decreased expression in 8-month-old *Fmn2*$^{-/-}$ mice (compared to wild-type mice) and were increased after Vorinostat treatment. In line with the gene expression data, we observed decreased H4K12ac in 8-month-old *Fmn2*$^{-/-}$ mice when compared *Fmn2*$^{+/+}$ animals (one-way ANOVA revealed a significant group difference for *Atp5d*, $P = 0.003$; *Ndufb7*, $P = 0.005$, and *Uqcrq*, $P = 0.001$). *Post hoc* comparison revealed a significant difference when comparing H4K12ac levels in the placebo-treated *Fmn2*$^{-/-}$ versus *Fmn2*$^{-/-}$ Vorinostat-treated or wild-type mice for all three genes (*$P < 0.05$; **$P < 0.01$; $n = 5$/group).

B Quantification of ChIP analysis showing H4K12ac at the promoter regions of three selected genes that showed decreased expression in 3-month-old *Fmn2*$^{-/-}$_APP mice (compared to 3-month-old APP mice) and were increased after Vorinostat treatment. One-way ANOVA revealed a significant group difference for *Cacng6*, $P = 0.004$; *Cdh19*, $P = 0.004$, and *Htr5b*, $P = 0.017$. *Post hoc* comparison revealed a significant difference when comparing H4K12ac levels in the placebo-treated APP versus *Fmn2*$^{-/-}$_APP mice and placebo versus Vorinostat-treated *Fmn2*$^{-/-}$_APP mice for all three genes (*$P < 0.05$; **$P < 0.01$; $n = 5$/group).

Data information: Error bars indicate SEM.

© 2017 The Authors

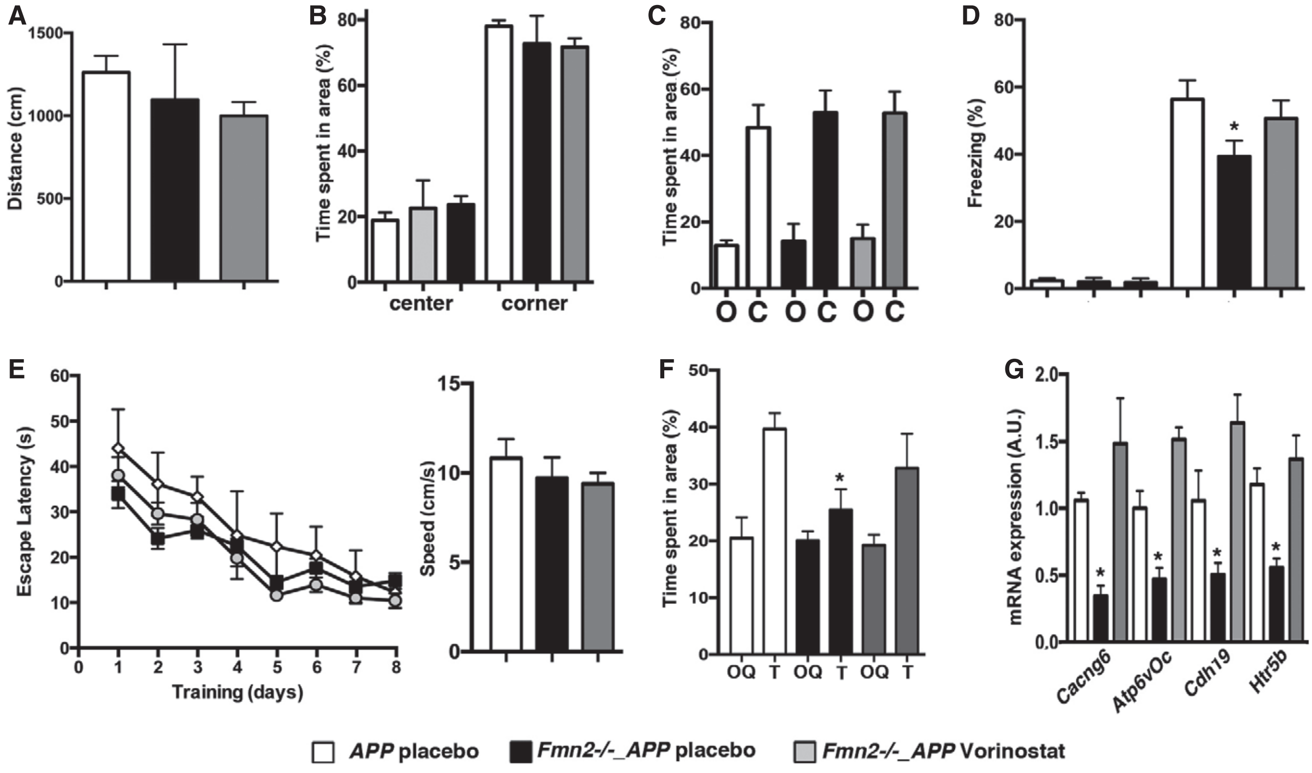

Figure EV5. Cognitive decline in 3-month-old *Fmn2*$^{-/-}$_APPPS1-21 mice is rescued by the HDAC inhibitor Vorinostat.

A The total distance traveled during a 5-min open-field exposure was similar amongst groups (n = 7/group).

B The time spent in the center or the corners during a 5-min open-field exposure was similar amongst groups (n = 7/group).

C The time spent in the open arms (o) and the closed arms (c) of an elevated plus maze was similar amongst groups (n = 9/group).

D One-way ANOVA revealed a significant difference in freezing behavior during the memory test (n = 12/group; F = 8.975, P = 0.0006). *Post hoc* analysis revealed increased freezing behavior when comparing the *Fmn2*$^{-/-}$_APP Vorinostat to the *Fmn2*$^{-/-}$_APP placebo group (*P = 0.139).

E Left panel: The escape latency in the water maze test was similar amongst groups (n = 12/group). Right panel: Swim speed was not altered amongst groups.

F Mice of the *Fmn2*$^{-/-}$_APP Vorinostat group spent significantly more time in the target quadrant (T) compared to the other quadrants (OQ). Please note that the values for OQ are presented as average of all three other quadrants (*P < 0.03, *post hoc* analysis, n = 12/group).

G The expression of selected genes affected in *Fmn2*$^{-/-}$_APP mice was increased when comparing the Vorinostat group to the placebo group (*P < 0.05; t-test, n = 4/group).

Data information: Error bars indicate SEM.

 © 2017 The Authors
